# Supplementary material for: Plant Phenology Supports the Multi-emergence Hypothesis for Ebola Spillover Events
Source: Ecohealth. 2017 Nov 13;15(3):497–508. doi: 10.1007/s10393-017-1288-z (PMC6245028; doi:10.1007/s10393-017-1288-z)
Supplement: Supplementary file 1 — Supplementary material 1 (DOCX 2444 kb) [file 10393_2017_1288_MOESM1_ESM.docx]

**Supplementary Material**

To the manuscript

**Plant phenology supports the eco-environmental hypothesis for Ebola spillover events**

**Contains:**

16 Supplementary Tables

2 Supplementary Figures

**Supplementary Table 1.** Principal component analysis of climate variables (annual averages, 1970-2012). Factor loadings in varimax-raw rotated coordinate system. Marked loadings are >0.7. Expl. Var - Explained Variance. Prp. Totl. Proportion of total explained variance.

|  | **YR_Clim PC1** | **YR_Clim PC2** | **YR_Clim PC3** | **YR_Clim PC4** | **YR_Clim PC5** |
| --- | --- | --- | --- | --- | --- |
| Avg normalized departure Apr-Oct rainfall departure | 0.4433 | -0.0117 | -0.2872 | -0.2173 | -0.4947 |
| 10 year avg rainfall Kibale Uganda | 0.1072 | 0.1887 | 0.6408 | -0.6188 | -0.0816 |
| Avg monthly min temp C Kibale Uganda | 0.0568 | -0.0348 | 0.0176 | **0.8920** | 0.1754 |
| Avg monthly max temp [C] Kibale Uganda | 0.4995 | -0.0212 | 0.3577 | -0.6011 | 0.2950 |
| DT90 | 0.0243 | **0.8090** | -0.2807 | 0.0387 | 0.1998 |
| DP01 | -0.3169 | -0.0697 | **-0.8069** | 0.0671 | 0.2789 |
| DP05 | -0.5887 | -0.0379 | -0.6789 | 0.0730 | 0.2873 |
| DP10 | **-0.7990** | -0.0284 | -0.4284 | 0.0766 | 0.2098 |
| CLDD | 0.0752 | 0.0030 | **-0.8814** | 0.0623 | 0.0850 |
| EMNT | 0.3043 | 0.1309 | 0.3591 | -0.1861 | **-0.7207** |
| EMXP | **-0.8958** | -0.0646 | 0.1381 | -0.0353 | 0.1108 |
| EMXT | 0.1476 | **0.8920** | -0.1057 | 0.0260 | -0.0722 |
| MMNT | 0.2495 | 0.3694 | 0.3611 | 0.0030 | **-0.7434** |
| MMXT | 0.0077 | **0.7782** | 0.3898 | -0.1697 | -0.3110 |
| MNTM | 0.0161 | **0.7853** | 0.3954 | -0.1011 | -0.3707 |
| TPCP | **-0.9226** | -0.1031 | -0.1401 | 0.0430 | 0.1225 |
| **Expl.Var** | **3.3830** | **2.8855** | **3.3899** | **1.6857** | **1.9594** |
| **Prp.Totl** | **0.2114** | **0.1803** | **0.2119** | **0.1054** | **0.1225** |

**Supplementary Table 2.** Eigenvalues of principal component analysis of climate variables (annual averages, 1970-2012).

|  | **Eigenvalue** | **% Total variance** | **Cumulative Eigenvalue** | **Cumulative %** |
| --- | --- | --- | --- | --- |
| **YR_Clim PC1** | 6.1967 | 38.7296 | 6.1967 | 38.7296 |
| **YR_Clim PC2** | 2.5862 | 16.1640 | 8.7830 | 54.8936 |
| **YR_Clim PC3** | 2.1760 | 13.5998 | 10.9589 | 68.4934 |
| **YR_Clim PC4** | 1.3266 | 8.2915 | 12.2856 | 76.7849 |
| **YR_Clim PC5** | 1.0180 | 6.3623 | 13.3035 | **83.1472** |

**Supplementary Table 3.** Principal component analysis for of phenology variables (annual averages, 1970-2012). Factor loadings in Varimax-raw rotated coordinate system. Marked loadings are >0.7. Expl. Var - Explained Variance. Prp. Totl. Proportion of total explained variance.

|  | **YR_Pheno PC1** | **YR_Pheno PC2** |
| --- | --- | --- |
| Proportion of population fruiting in Kibale NP Uganda | -0.0651 | **0.8673** |
| IC1 NDVI anomaly (AUG-Nov) | **0.8919** | -0.2085 |
| IC2 NDVI anomaly(Jul-Dec) | **0.7628** | 0.4047 |
| Flowering Anomalies Lope Gabon | 0.1902 | 0.5240 |
| **Expl.Var** | **1.4178** | **1.2341** |
| **Prp.Totl** | **0.3544** | **0.3085** |

**Supplementary Table 4.** Eigenvalues of principal component analysis for phenology variables (annual averages, 1970-2012).

|  | **Eigenvalue** | **% Total variance** | **Cumulative Eigenvalue** | **Cumulative %** |
| --- | --- | --- | --- | --- |
| **YR_Pheno PC1** | 1.5156 | 37.8902 | 1.5156 | 37.8902 |
| **YR_Pheno PC2** | 1.1363 | 28.4065 | 2.6519 | 66.2968 |

**Supplementary Table 5**. Principal component analysis of climate variables (monthly averages across all years). Table shows factor loadings in Varimax-raw rotated coordinate system. Factor loadings over 7 are marked in bold. Expl. Var - Explained Variance. Prp. Totl. Proportion of total explained variance.

|  | **S_Climate PC1** | **S_Climate PC2** | **S_Climate PC3** |
| --- | --- | --- | --- |
| Monthly inner-annual linear trends of rainfall | 0.2729 | 0.3199 | 0.0839 |
| Monthly inner-annual linear trends of rainfall | 0.2246 | 0.0955 | **0.8547** |
| Avg_Mo Rainfall Humid tropical Africa | -0.0398 | 0.1364 | **-0.9492** |
| Avg_Mo Temp Central Africa | 0.3742 | **0.7947** | 0.4255 |
| Estimated inter-annual trends of rainfall | 0.1579 | **-0.9410** | 0.1264 |
| CLDD | **0.7865** | 0.4158 | 0.3886 |
| EMNT | 0.6393 | 0.0493 | **0.7142** |
| EMXP | **0.9548** | -0.0832 | 0.1985 |
| EMXT | 0.5951 | 0.4080 | 0.6726 |
| MMNT | 0.6469 | 0.2127 | 0.6736 |
| MMXT | 0.6385 | 0.2786 | **0.7049** |
| MNTM | 0.6457 | 0.2471 | **0.7078** |
| TPCP | **0.9583** | -0.0479 | 0.1307 |
| **Expl.Var** | **4.7458** | **2.1819** | **4.4569** |
| **Prp.Totl** | **0.3651** | **0.1678** | **0.3428** |

**Supplementary Table 6.** Eigenvalues of principal component analysis for climate variables (monthly averages across all years).

|  | **Eigenvalue** | **% Total variance** | **Cumulative Eigenvalue** | **Cumulative %** |
| --- | --- | --- | --- | --- |
| **S_Climate PC1** | 8.3124 | 63.9418 | 8.3124 | 63.9418 |
| **S_Climate PC2** | 1.7261 | 13.2776 | 10.0385 | 77.2195 |
| **S_Climate PC3** | 1.3463 | 10.3558 | 11.3848 | 87.5753 |

**Supplementary Table 7**. Principal component analysis of phenology variables (monthly averages across all years). Table shows factor loadings in Varimax-raw rotated coordinate system. Factor loadings over 7 are marked in bold. Expl. Var - Explained Variance. Prp. Totl. Proportion of total explained variance.

|  | **S_Phenology PC1** | **S_Phenology PC2** | **S_Phenology PC3** | **S_Phenology PC4** | **S_Phenology PC5** | **S_Phenology PC6** |
| --- | --- | --- | --- | --- | --- | --- |
| Fruit Index Chimpanzee Primary Forest | 0.1184 | **-0.8826** | 0.1463 | 0.2235 | -0.2533 | 0.0868 |
| Fruit Index Chimpanzee Secondary Forest | -0.1308 | 0.1372 | -0.2216 | 0.0248 | 0.1071 | **-0.9250** |
| Fruit Index Gorilla Primary Forest | 0.0174 | -0.0521 | **0.8438** | 0.1127 | -0.1476 | -0.2242 |
| Fruit Index Gorilla Secondary Forest | **0.7283** | -0.0737 | 0.5079 | 0.3475 | 0.0804 | 0.0059 |
| *Allophylus* % | 0.3792 | **-0.7989** | -0.0812 | 0.3180 | 0.2552 | -0.0258 |
| *Bridelia bridelifolia* % | 0.4386 | 0.3048 | 0.5177 | -0.3162 | 0.3543 | -0.3858 |
| *Cassipourea ruwenzoriensis* % | **0.8794** | 0.2425 | -0.1937 | -0.0122 | 0.2717 | 0.0221 |
| *Diospyros honleana* % | 0.3445 | **-0.8236** | -0.0436 | 0.2262 | 0.3510 | -0.0805 |
| *Ekebergia capensis* % | **0.9179** | -0.1145 | 0.2378 | 0.1812 | 0.1528 | 0.0928 |
| *Ficus oreodryadum* % | 0.1425 | -0.1852 | 0.0987 | **0.9237** | -0.1400 | -0.0476 |
| *Ficus thonningii* % | -0.1121 | -0.2976 | -0.3284 | **0.8084** | -0.1078 | 0.1488 |
| *Maesa lanceolata* % | 0.4903 | 0.3965 | 0.3518 | -0.0667 | 0.4213 | -0.5211 |
| *Myrianthus holstii* % | **0.8477** | 0.1948 | 0.1994 | -0.2014 | 0.2155 | -0.2657 |
| *Newtonia buchanani* % | -0.4914 | -0.5157 | 0.1445 | -0.3537 | -0.4775 | 0.2340 |
| *Psychotria palustris* % | 0.1952 | 0.4511 | 0.3180 | -0.5283 | 0.3042 | -0.5275 |
| *Syzygium parvifolium* % | **-0.1775** | **-0.8984** | -0.1085 | -0.0123 | 0.0446 | 0.3114 |
| Community bearing fruit | **-0.9339** | 0.1298 | -0.2172 | -0.1233 | -0.0531 | 0.0859 |
| Counts of # of species w/ peak fruiting times by month | **-0.8035** | 0.3717 | -0.0669 | 0.0700 | 0.2190 | -0.0981 |
| % of trees with ripe fruit Kibale NP | 0.2001 | 0.2538 | 0.5340 | 0.2584 | -0.4942 | 0.4221 |
| Proportion of trees flowering at Goualougo | -0.3128 | 0.1604 | -0.3478 | 0.0334 | **-0.8312** | 0.0114 |
| Proportion of trees flowering in Lope Gabon | **-0.8113** | 0.2852 | -0.3571 | 0.2429 | 0.0012 | -0.1206 |
| Proportion of trees flowering at Okapi | -0.0610 | 0.0449 | 0.1515 | 0.2442 | **-0.8973** | 0.1461 |
| Proportion of trees with ripe fruit Goualougo | 0.0512 | 0.0829 | **0.8532** | -0.0245 | 0.0857 | 0.3732 |
| Proportion of trees with ripe fruit in Lope | -0.5800 | 0.1742 | -0.7232 | 0.1175 | -0.0415 | 0.0729 |
| Proportion of trees with ripe fruit in Okapi | 0.3029 | 0.0475 | **0.8974** | -0.2175 | 0.0785 | 0.0975 |
| **Expl.Var** | **6.8156** | **4.4798** | **4.7914** | **2.7679** | **2.8997** | **2.4768** |
| **Prp.Totl** | **0.2621** | **0.1723** | **0.1843** | **0.1065** | **0.1115** | **0.0953** |

**Supplementary Table 8.** Eigenvalues of principal component analysis for phenology variables (monthly averages across all years).

|  | **Eigenvalue** | **% Total variance** | **Cumulative Eigenvalue** | **Cumulative %** |
| --- | --- | --- | --- | --- |
| **S_Phenology PC1** | 9.3194 | 35.8439 | 9.3194 | 35.8439 |
| **S_Phenology PC2** | 5.8795 | 22.6134 | 15.1989 | 58.4573 |
| **S_Phenology PC3** | 3.7239 | 14.3228 | 18.9228 | 72.7801 |
| **S_Phenology PC4** | 2.3836 | 9.1676 | 21.3064 | 81.9477 |
| **S_Phenology PC5** | 1.6529 | 6.3573 | 22.9593 | 88.3050 |
| **S_Phenology PC6** | 1.2719 | 4.8921 | 24.2312 | 93.1971 |

**Supplementary Table 9.** Climate principal component analysis for monthly 1994-2002 data partition. Factor loadings in Varimax-raw rotated coordinate system. Marked loadings are >.700000. Expl. Var - Explained Variance. Prp. Totl. Proportion of total explained variance.

|  | **M_Climate PC1** | **M_Climate PC2** | **M_Climate PC3** |
| --- | --- | --- | --- |
| CLDD_Makokou | 0.5044 | 0.0990 | -0.1739 |
| EMNT_Makokou | -0.0387 | -0.0088 | **0.9354** |
| EMXP_Makokou | 0.0297 | **0.9748** | 0.0359 |
| EMXT_Makokou | **0.9160** | 0.0621 | -0.0665 |
| MMNT_Makokou | 0.5312 | 0.0331 | **0.7484** |
| MMXT_Makokou | **0.9031** | 0.0688 | 0.1353 |
| MNTM_Makokou | **0.9230** | 0.0792 | 0.2017 |
| TPCP_Makokou | 0.1040 | **0.9702** | -0.0272 |
| Expl.Var | 3.0563 | 1.9173 | 1.5308 |
| Prp.Totl | 0.3820 | 0.2397 | 0.1914 |

**Supplementary Table 10.**  Eigenvalues of principal component analysis for climate variables (monthly values for 1994-2002).

|  | **Eigenvalue** | **% Total variance** | **Cumulative Eigenvalue** | **Cumulative %** |
| --- | --- | --- | --- | --- |
| **M_Climate PC1** | 3.3202 | 41.5020 | 3.3202 | 41.5020 |
| **M_Climate PC2** | 1.8291 | 22.8641 | 5.1493 | 64.3661 |
| **M_Climate PC3** | 1.3552 | 16.9394 | 6.5044 | 81.3055 |

**Supplementary Table 11.** Principal component analysis for fruiting variables (monthly 1994-2002 data partition). Factor loadings in varimax raw rotated coordinate system. Marked loadings are >.700000. Expl. Var - Explained Variance. Prp. Totl. Proportion of total explained variance.

|  | **M_Fruit PC1** | **M_Fruit PC2** |
| --- | --- | --- |
| Fruit Index Chimpanzee Primary Forest | 0.3300 | 0.6476 |
| Fruit Index Chimpanzee Secondary Forest | 0.5118 | 0.4491 |
| Fruit Index Gorilla Primary Forest | **0.8035** | -0.0331 |
| Fruit Index Gorilla Secondary Forest | **0.9039** | 0.0219 |
| % of trees with ripe fruit Kibale | 0.1354 | **-0.8621** |
| Expl.Var | 1.8519 | 1.3658 |
| Prp.Totl | 0.3704 | 0.2732 |

**Supplementary Table 12.**  Eigenvalues of principal component analysis for fruiting variables (monthly values for 1994-2002).

|  | **Eigenvalue** | **% Total**  **variance** | **Cumulative Eigenvalue** | **Cumulative %** |
| --- | --- | --- | --- | --- |
| **M_Fruit PC1** | 2.0107 | 40.2136 | 2.0107 | 40.2136 |
| **M_Fruit PC2** | 1.2070 | 24.1409 | 3.2177 | 64.3544 |

**Supplementary Table 13.** Principal component analysis for single species fruiting at Kahuzi-Biega National Park (monthly 1994-2002 data partition). Factor loadings in varimax raw rotated coordinate system. Marked loadings are >.700000. Expl. Var - Explained Variance. Prp. Totl. Proportion of total explained variance.

|  | **M_Plant PC1** | **M_Plant PC2** | **M_Plant PC3** | **M_Plant PC4** | **M_Plant PC5** |
| --- | --- | --- | --- | --- | --- |
| *Allophylus sp.* | -0.0844 | 0.1602 | 0.1150 | 0.4437 | 0.6891 |
| *Bridelia bridelifolia* | **0.8129** | 0.1206 | 0.1947 | 0.1109 | -0.1222 |
| *Cassipourea ruwenzoriensis* | 0.0107 | 0.1379 | 0.0027 | 0.7176 | -0.2829 |
| *Diospyros honleana* | -0.1806 | 0.1674 | 0.6507 | 0.0846 | 0.4619 |
| *Ekebergia capensis* | 0.0902 | 0.1134 | **0.0693** | **0.8270** | 0.0303 |
| *Ficus oreodryadum* | -0.0030 | **0.7453** | -0.3988 | 0.0936 | 0.0561 |
| *Ficus thonningii* | 0.0673 | **0.8642** | 0.1740 | 0.0366 | 0.0218 |
| *Maesa lanceolata* | **0.7061** | 0.2418 | -0.2716 | 0.3523 | 0.0206 |
| *Myrianthus holstii* | 0.4152 | -0.2531 | -0.2518 | 0.6371 | 0.1146 |
| *Newtonia buchananii* | -0.3148 | 0.1493 | **-0.7066** | 0.0574 | 0.1865 |
| *Psychotria palustris* | **0.8865** | -0.1158 | 0.0581 | -0.0398 | -0.0436 |
| *Syzygium parvifolium* | -0.0372 | -0.0425 | -0.0626 | -0.2439 | **0.8485** |
| Expl.Var | 2.2708 | 1.5624 | 1.3124 | 2.0197 | 1.5580 |
| Prp.Totl | 0.1892 | 0.1302 | 0.1094 | 0.1683 | 0.1298 |

**Supplementary Table 14.** Eigenvalues of principal component analysis for variables describing fruiting of different species of plants (monthly values for 1994-2002).

|  | **Eigenvalue** | **% Total variance** | **Cumulative Eigenvalue** | **Cumulative%** |
| --- | --- | --- | --- | --- |
| **M_Plant PC1** | 2.8275 | 23.5629 | 2.8275 | 23.5629 |
| **M_Plant PC2** | 1.9852 | 16.5433 | 4.8127 | 40.1061 |
| **M_Plant PC3** | 1.4789 | 12.3243 | 6.2916 | 52.4304 |
| **M_Plant PC4** | 1.2932 | 10.7764 | 7.5848 | 63.2068 |
| **M_Plant PC5** | 1.1385 | 9.4877 | 8.7233 | 72.6945 |

**Supplementary Table 15.** Neural Network models for time series regression of annual averages (1970-2012). 500 Networks each were run with (i) Climate, (ii) Phenology, and (iii) Climate + Phenology as sets of input variables to predict number of spillover events. For visual performance of each set of input variables in predicting the number of human + animal spillover events in the model dataset, see Supplementary Figure 2.

| **Input Vars** | **Climate + Phenology** | **Phenology** | **Climate** |
| --- | --- | --- | --- |
| **No. of best 5 retained networks** | 5 | 2 | 4 |
| **Best network name** | MLP 7-10-1 | MLP 2-8-1 | MLP 5-9-1 |
| **Training perf.** | 0.7676 | 0.6975 | 0.8255 |
| **Test perf.** | 0.993 | 0.8622 | 0.9513 |
| **Validation perf.** | 0.9487 | 0.9486 | 0.9487 |
| **Training error** | 0.2745 | 0.1192 | 0.2006 |
| **Test error** | 6.3027 | 5.9242 | 5.5365 |
| **Validation error** | 1.1829 | 0.7086 | 0.842521 |
| **Training algorithm** | BFGS 11 | BFGS 69 | BFGS 28 |
| **Error function** | SOS | SOS | SOS |
| **Hidden activation** | Exponential | Tanh | Tanh |
| **Output activation** | Logistic | Identity | Identity |

**Supplementary Table 16.** T-test for dependent samples. Marked differences are significant at p < .05000. Test between observed animal and human spillover events, and predicted values from Neural Network models in cross-validation data set.

|  | **Mean** | **Std.Dv.** | **N** | **Diff.** | **Std.Dv.Diff.** | **t** | **df** | **p** | **Confidence**  **-95%** | **Confidence**  **+95%** |
| --- | --- | --- | --- | --- | --- | --- | --- | --- | --- | --- |
| Observed spillovers | 1.3500 | 3.2163 |  |  |  |  |  |  |  |  |
| Clim +Pheno  predicted  spillovers | 0.7696 | 1.1830 | 20 | 0.5804 | 2.4316 | 1.0675 | 19 | 0.2991 | -0.5576 | 1.7185 |
| Pheno  predicted  spillovers | 1.2180 | 2.5928 | 20 | 0.1320 | 1.5131 | 0.3902 | 19 | 0.7007 | -0.5761 | 0.8402 |
| Clim  predicted  spillovers | 0.7288 | 1.1233 | 20 | 0.6212 | 2.6623 | 1.0434 | 19 | 0.3098 | -0.6248 | 1.8671 |

**Supplementary Figure 1.** Interannual variation in climate (red) and phenology (green) variables, as well as recorded human+other mammal Ebola spillover events (blue).


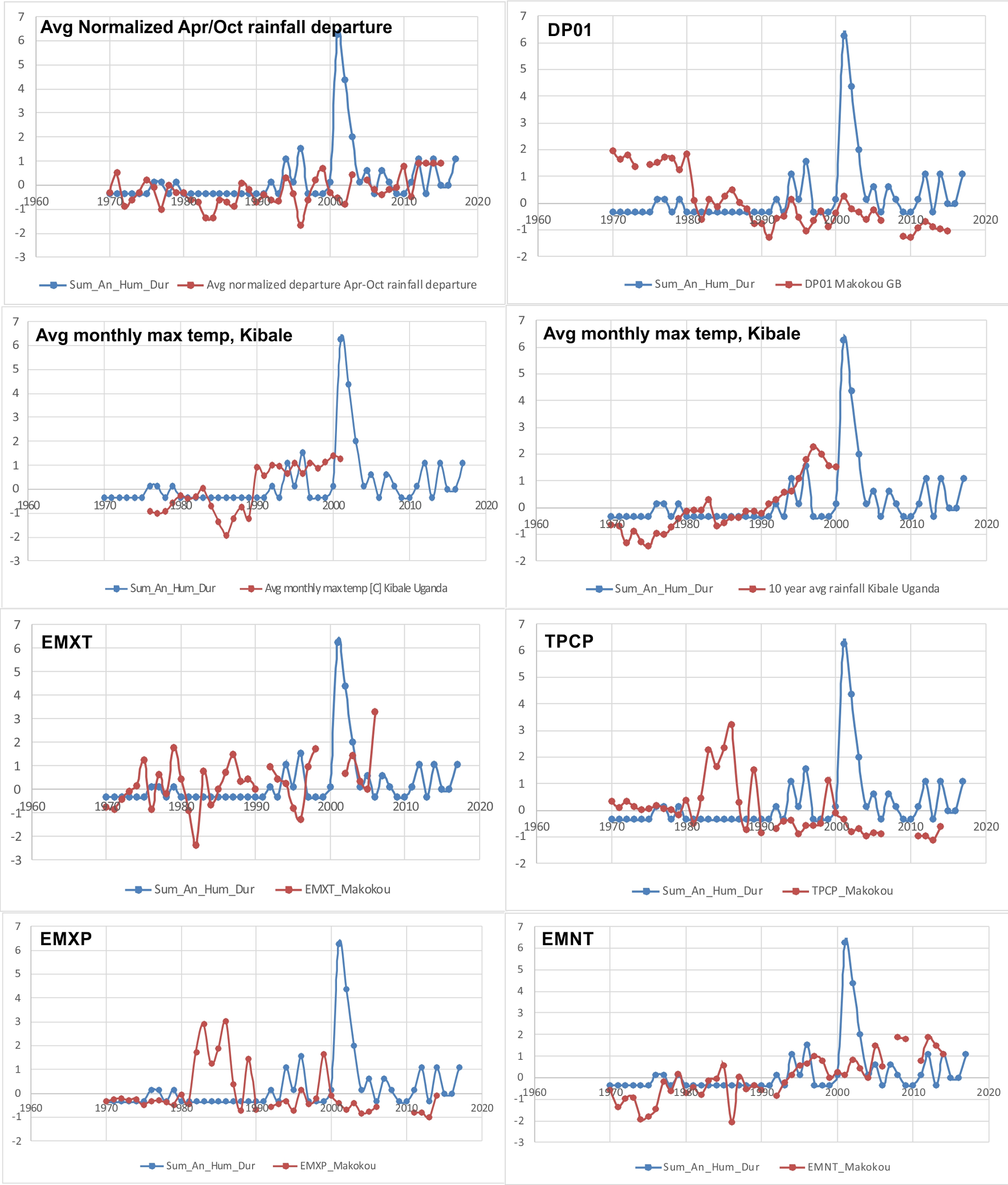


**Supplementary Figure 1 (ctd.).** Interannual variation in climate (red) and phenology (green) variables, as well as recorded human+other mammal Ebola spillover events (blue).


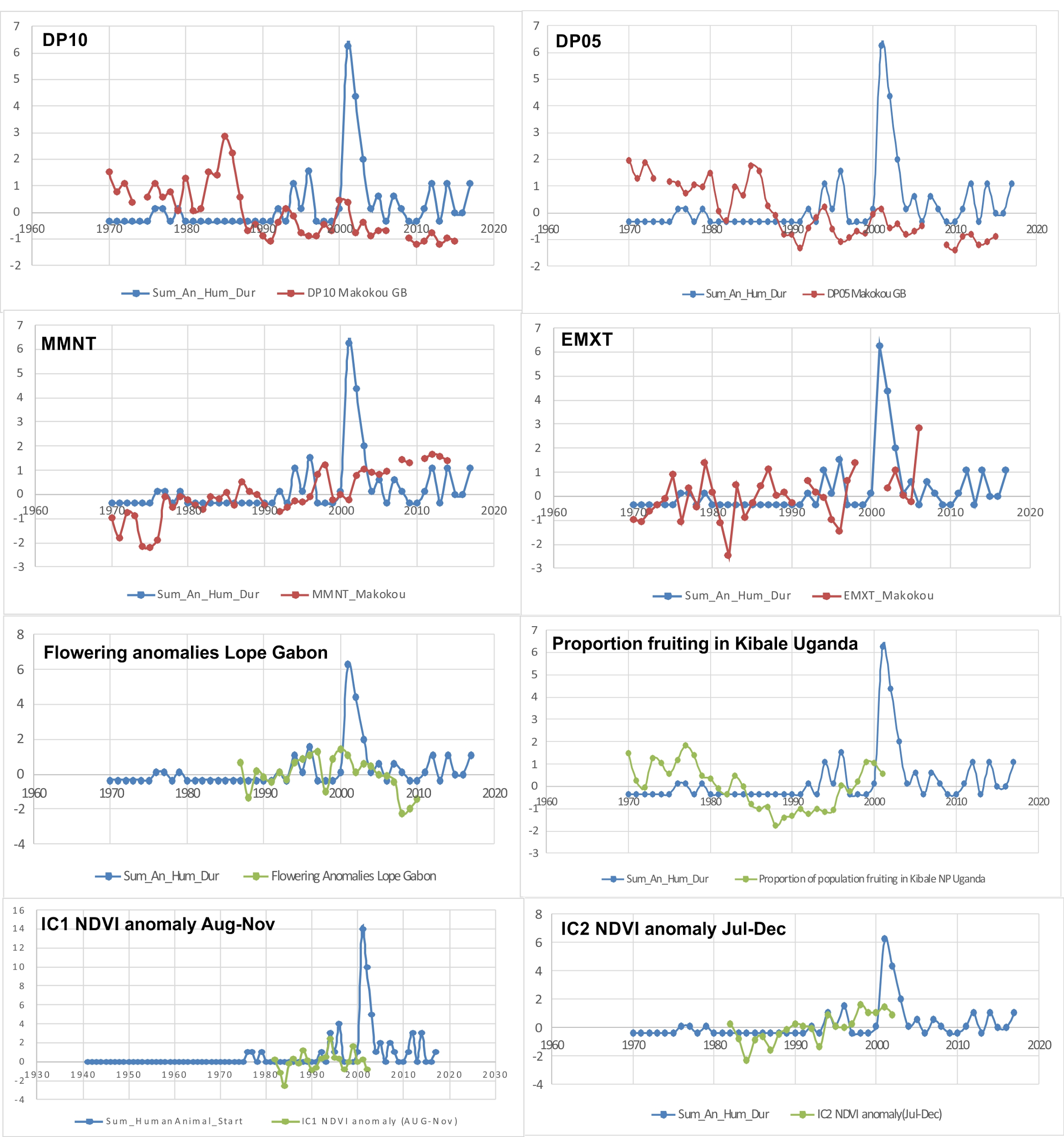


**Supplementary Figure 2.** Neural Network training dataset average annual 1970-2012 partition of climate and phenology variables. Shown are Time series predictions for the observed number of human and other mammal spillover, vs. the predicted numbers from best retained model (500 network iterations). One step was used as input, and one step predicted ahead. Samples included train, test, and validation data points. Panel a) climate and phenology PCs as input variables; b) only phenology PCs as input variables; c) only climate PCs as input variables.
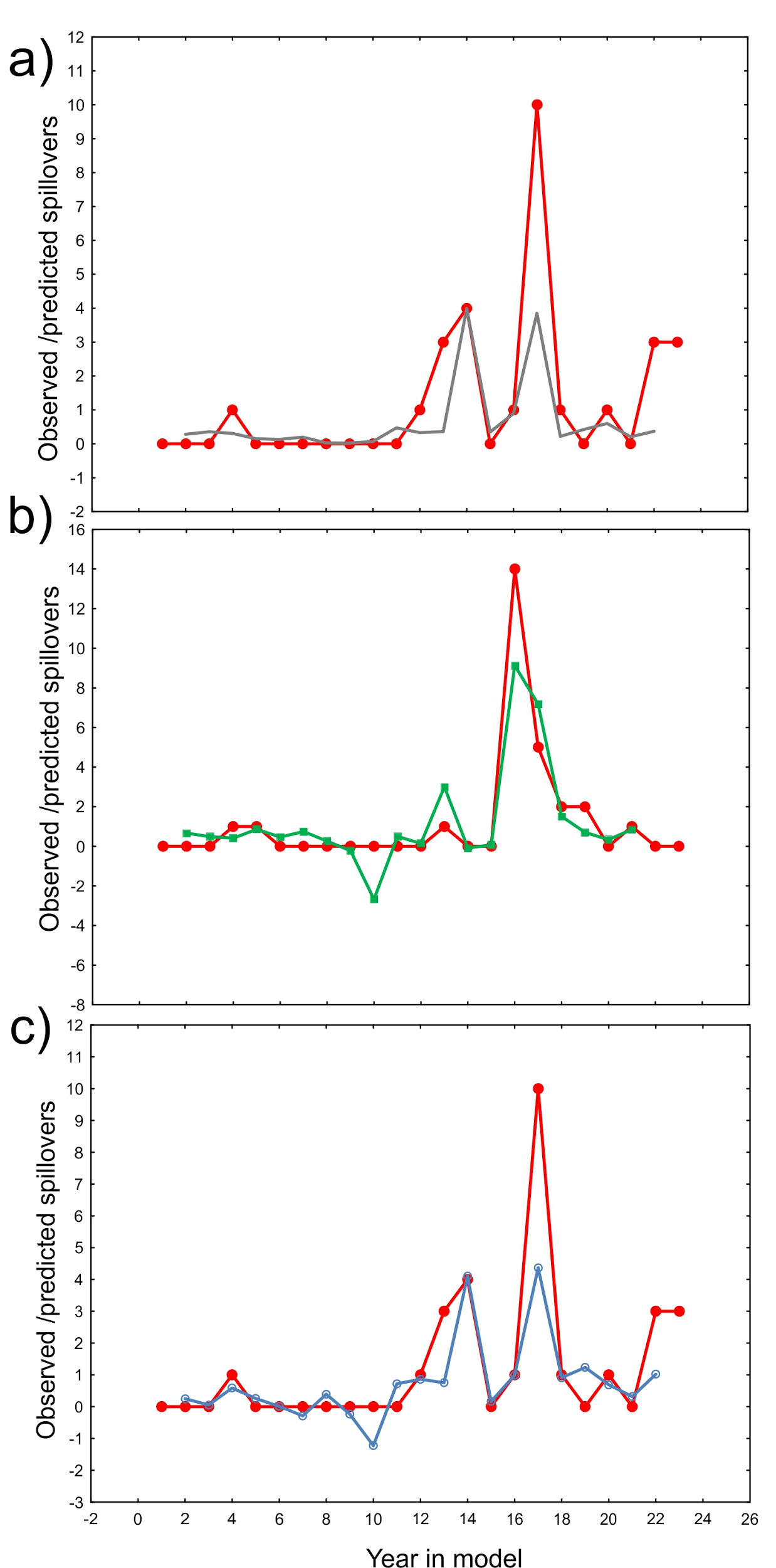


**Supplementary Appendix 1.** Climate input variables, Neural Networks, best model of 500, .xml format (in Predictive Model Markup Language PMML).

<?xml version="1.0" encoding="UTF-8"?>
<PMML version="3.0"><Header copyright="Copyright 1984-2016 Dell Inc. All Rights Reserved."><Application name="STATISTICA Automated Neural Networks (SANN)" version="2.0"/></Header><DataDictionary numberOfFields="6"><DataField name="Sum_Humanother mammal_Start" optype="continuous"/><DataField name="ClimPC1" optype="continuous"/><DataField name="ClimPC2" optype="continuous"/><DataField name="ClimPC3" optype="continuous"/><DataField name="ClimPC4" optype="continuous"/><DataField name="ClimPC5" optype="continuous"/></DataDictionary><NeuralNetwork modelName="SUMAVGYR_M_MLP 5-9-1" functionName="timeseries" stepsUsed="1" stepsAhead="1"><MiningSchema><MiningField name="Sum_Humanother mammal_Start" usageType="predicted"/><MiningField name="ClimPC1" lowValue="-3.292020" highValue="1.777644"/><MiningField name="ClimPC2" lowValue="-4.789920" highValue="2.153318"/><MiningField name="ClimPC3" lowValue="-0.983662" highValue="2.798979"/><MiningField name="ClimPC4" lowValue="-2.010414" highValue="1.637324"/><MiningField name="ClimPC5" lowValue="-2.259344" highValue="2.010512"/></MiningSchema><NeuralInputs numberOfInputs="5"><NeuralInput id="0"><DerivedField><NormContinuous field="ClimPC1"><LinearNorm orig="-3.29202040252129e+000" norm="0.000000"/><LinearNorm orig="1.77764378562846e+000" norm="1.000000"/></NormContinuous></DerivedField></NeuralInput><NeuralInput id="1"><DerivedField><NormContinuous field="ClimPC2"><LinearNorm orig="-4.78991958973482e+000" norm="0.000000"/><LinearNorm orig="2.15331815820348e+000" norm="1.000000"/></NormContinuous></DerivedField></NeuralInput><NeuralInput id="2"><DerivedField><NormContinuous field="ClimPC3"><LinearNorm orig="-9.83661600897224e-001" norm="0.000000"/><LinearNorm orig="2.79897885015328e+000" norm="1.000000"/></NormContinuous></DerivedField></NeuralInput><NeuralInput id="3"><DerivedField><NormContinuous field="ClimPC4"><LinearNorm orig="-2.01041447516216e+000" norm="0.000000"/><LinearNorm orig="1.63732366055998e+000" norm="1.000000"/></NormContinuous></DerivedField></NeuralInput><NeuralInput id="4"><DerivedField><NormContinuous field="ClimPC5"><LinearNorm orig="-2.25934363043270e+000" norm="0.000000"/><LinearNorm orig="2.01051219276550e+000" norm="1.000000"/></NormContinuous></DerivedField></NeuralInput></NeuralInputs><NeuralLayer numberOfNeurons="9" activationFunction="tanh"><Neuron id="5" bias="-4.57544870168567e-001"><Con from="0" weight="-6.42630247824497e-001"/><Con from="1" weight="-1.10728745571426e-001"/><Con from="2" weight="-8.55073950638736e-001"/><Con from="3" weight="2.58715740102447e-001"/><Con from="4" weight="-2.11135476379042e-001"/></Neuron><Neuron id="6" bias="7.02498846555938e-001"><Con from="0" weight="-8.59957997906533e-001"/><Con from="1" weight="-1.68230379687652e-001"/><Con from="2" weight="4.56064016293882e-001"/><Con from="3" weight="7.02594424053140e-001"/><Con from="4" weight="3.65679254662808e-001"/></Neuron><Neuron id="7" bias="1.49844880697107e+000"><Con from="0" weight="1.79602327720144e+000"/><Con from="1" weight="8.02420028961292e-001"/><Con from="2" weight="3.26425072060314e-001"/><Con from="3" weight="-1.46360792592624e+000"/><Con from="4" weight="7.41448550105352e-001"/></Neuron><Neuron id="8" bias="-5.08933370431126e-001"><Con from="0" weight="6.51823853952154e-001"/><Con from="1" weight="-9.26636841471553e-001"/><Con from="2" weight="-1.19971392722349e+000"/><Con from="3" weight="-2.69417920371521e+000"/><Con from="4" weight="-4.49765302604416e-001"/></Neuron><Neuron id="9" bias="-4.20121916906816e-001"><Con from="0" weight="5.07182495954279e-001"/><Con from="1" weight="-9.04012017007920e-001"/><Con from="2" weight="-7.64914818328622e-001"/><Con from="3" weight="-2.13999403668308e+000"/><Con from="4" weight="-4.02384099732223e-001"/></Neuron><Neuron id="10" bias="6.54236572280325e-001"><Con from="0" weight="4.54402353771700e-001"/><Con from="1" weight="4.03949057122599e-001"/><Con from="2" weight="4.65460104907936e-001"/><Con from="3" weight="1.32572725117419e-001"/><Con from="4" weight="4.15749462634562e-001"/></Neuron><Neuron id="11" bias="8.34279934161979e-001"><Con from="0" weight="7.70310499816946e-001"/><Con from="1" weight="5.96303926650555e-001"/><Con from="2" weight="8.71596812835657e-001"/><Con from="3" weight="3.23078692271286e-001"/><Con from="4" weight="6.26097522169381e-001"/></Neuron><Neuron id="12" bias="-4.56618639288198e-002"><Con from="0" weight="1.19675865898896e-001"/><Con from="1" weight="-1.59753558016023e-001"/><Con from="2" weight="2.13677625761914e-002"/><Con from="3" weight="-8.84860290595226e-001"/><Con from="4" weight="-7.52903339758650e-002"/></Neuron><Neuron id="13" bias="5.21108244611361e-003"><Con from="0" weight="1.99841236364020e-001"/><Con from="1" weight="-1.67795829758265e-001"/><Con from="2" weight="2.10127539086111e-001"/><Con from="3" weight="-1.12139639076574e+000"/><Con from="4" weight="-1.62936901691171e-001"/></Neuron></NeuralLayer><NeuralLayer numberOfNeurons="1" activationFunction="identity"><Neuron id="14" bias="4.85211476116981e-001"><Con from="5" weight="-1.09238665448429e+000"/><Con from="6" weight="-1.16155099250744e+000"/><Con from="7" weight="-1.67059180220083e+000"/><Con from="8" weight="1.49759756092075e+000"/><Con from="9" weight="7.27880461255915e-001"/><Con from="10" weight="9.39739575311965e-001"/><Con from="11" weight="2.10598728120108e+000"/><Con from="12" weight="-4.14234553945588e-001"/><Con from="13" weight="-2.55257043275488e-001"/></Neuron></NeuralLayer><NeuralOutputs numberOfOutputs="1"><NeuralOutput outputNeuron="14"><DerivedField optype="continuous"><NormContinuous field="Sum_Humanother mammal_Start"><LinearNorm orig="0.00000000000000e+000" norm="0.00000000000000e+000"/><LinearNorm orig="4.00000000000000e+000" norm="1.00000000000000e+000"/></NormContinuous></DerivedField></NeuralOutput></NeuralOutputs></NeuralNetwork></PMML>

**Supplementary Appendix 2.** Phenology input variables, Neural Networks, best model of 500, .xml format (in Predictive Model Markup Language PMML).

<?xml version="1.0" encoding="UTF-8"?>
<PMML version="3.0"><Header copyright="Copyright 1984-2016 Dell Inc. All Rights Reserved."><Application name="STATISTICA Automated Neural Networks (SANN)" version="2.0"/></Header><DataDictionary numberOfFields="3"><DataField name="Observed_Sum_HAStart" optype="continuous"/><DataField name="PhenoPC1" optype="continuous"/><DataField name="PhenoPC2" optype="continuous"/></DataDictionary><NeuralNetwork modelName="SUMAVGYR_Cros_MLP 2-8-1" functionName="timeseries" stepsUsed="1" stepsAhead="1"><MiningSchema><MiningField name="Observed_Sum_HAStart" usageType="predicted"/><MiningField name="PhenoPC1" lowValue="-2.348446" highValue="2.969327"/><MiningField name="PhenoPC2" lowValue="-2.251694" highValue="2.067669"/></MiningSchema><NeuralInputs numberOfInputs="2"><NeuralInput id="0"><DerivedField><NormContinuous field="PhenoPC1"><LinearNorm orig="-2.34844552576411e+000" norm="0.000000"/><LinearNorm orig="2.96932663174580e+000" norm="1.000000"/></NormContinuous></DerivedField></NeuralInput><NeuralInput id="1"><DerivedField><NormContinuous field="PhenoPC2"><LinearNorm orig="-2.25169445319774e+000" norm="0.000000"/><LinearNorm orig="2.06766856691779e+000" norm="1.000000"/></NormContinuous></DerivedField></NeuralInput></NeuralInputs><NeuralLayer numberOfNeurons="8" activationFunction="tanh"><Neuron id="2" bias="-1.44015612723403e+000"><Con from="0" weight="-8.59048686000305e-001"/><Con from="1" weight="3.24099130764678e+000"/></Neuron><Neuron id="3" bias="-1.78772685013887e-001"><Con from="0" weight="-6.70885447742957e-001"/><Con from="1" weight="1.92140225620363e-001"/></Neuron><Neuron id="4" bias="-2.93053639275249e+000"><Con from="0" weight="-1.01556572047421e+000"/><Con from="1" weight="5.67866530060885e+000"/></Neuron><Neuron id="5" bias="-8.30477065007423e-002"><Con from="0" weight="-6.82429902277289e+000"/><Con from="1" weight="4.96570950057858e+000"/></Neuron><Neuron id="6" bias="4.38536770968002e-001"><Con from="0" weight="-1.51596615956532e+000"/><Con from="1" weight="-2.00350142115466e+000"/></Neuron><Neuron id="7" bias="-2.21877101128317e+000"><Con from="0" weight="-5.36112227885903e-001"/><Con from="1" weight="-5.33096409943744e-001"/></Neuron><Neuron id="8" bias="-6.49932141646218e-001"><Con from="0" weight="3.21158592447094e-001"/><Con from="1" weight="-8.86900934499459e-001"/></Neuron><Neuron id="9" bias="4.71791402984549e-001"><Con from="0" weight="-5.16909767259391e-001"/><Con from="1" weight="2.84156268049183e+000"/></Neuron></NeuralLayer><NeuralLayer numberOfNeurons="1" activationFunction="identity"><Neuron id="10" bias="3.49416414856537e-001"><Con from="2" weight="2.19617656908569e+000"/><Con from="3" weight="4.21190367770349e-001"/><Con from="4" weight="1.36624112960725e+000"/><Con from="5" weight="-2.70150010057860e+000"/><Con from="6" weight="2.23126552825783e+000"/><Con from="7" weight="-2.35102654812819e+000"/><Con from="8" weight="-5.88445772704538e-001"/><Con from="9" weight="-1.18981120573560e+000"/></Neuron></NeuralLayer><NeuralOutputs numberOfOutputs="1"><NeuralOutput outputNeuron="10"><DerivedField optype="continuous"><NormContinuous field="Observed_Sum_HAStart"><LinearNorm orig="0.00000000000000e+000" norm="0.00000000000000e+000"/><LinearNorm orig="2.00000000000000e+000" norm="1.00000000000000e+000"/></NormContinuous></DerivedField></NeuralOutput></NeuralOutputs></NeuralNetwork></PMML>

**Supplementary Appendix 3.** Phenology and climate input variables, Neural Networks, best model of 500, .xml format (in Predictive Model Markup Language PMML).

<?xml version="1.0" encoding="UTF-8"?>
<PMML version="3.0"><Header copyright="Copyright 1984-2016 Dell Inc. All Rights Reserved."><Application name="STATISTICA Automated Neural Networks (SANN)" version="2.0"/></Header><DataDictionary numberOfFields="8"><DataField name="Sum_Humanother mammal_Start" optype="continuous"/><DataField name="ClimPC1" optype="continuous"/><DataField name="ClimPC2" optype="continuous"/><DataField name="ClimPC3" optype="continuous"/><DataField name="ClimPC4" optype="continuous"/><DataField name="ClimPC5" optype="continuous"/><DataField name="PhenoPC1" optype="continuous"/><DataField name="PhenoPC2" optype="continuous"/></DataDictionary><NeuralNetwork modelName="SUMAVGYR_M_MLP 7-10-1" functionName="timeseries" stepsUsed="1" stepsAhead="1"><MiningSchema><MiningField name="Sum_Humanother mammal_Start" usageType="predicted"/><MiningField name="ClimPC1" lowValue="-3.292020" highValue="1.777644"/><MiningField name="ClimPC2" lowValue="-4.789920" highValue="2.153318"/><MiningField name="ClimPC3" lowValue="-0.983662" highValue="2.798979"/><MiningField name="ClimPC4" lowValue="-2.010414" highValue="1.637324"/><MiningField name="ClimPC5" lowValue="-2.259344" highValue="2.010512"/><MiningField name="PhenoPC1" lowValue="-5.410674" highValue="1.356991"/><MiningField name="PhenoPC2" lowValue="-1.666108" highValue="1.695266"/></MiningSchema><NeuralInputs numberOfInputs="7"><NeuralInput id="0"><DerivedField><NormContinuous field="ClimPC1"><LinearNorm orig="-3.29202040252129e+000" norm="0.000000"/><LinearNorm orig="1.77764378562846e+000" norm="1.000000"/></NormContinuous></DerivedField></NeuralInput><NeuralInput id="1"><DerivedField><NormContinuous field="ClimPC2"><LinearNorm orig="-4.78991958973482e+000" norm="0.000000"/><LinearNorm orig="2.15331815820348e+000" norm="1.000000"/></NormContinuous></DerivedField></NeuralInput><NeuralInput id="2"><DerivedField><NormContinuous field="ClimPC3"><LinearNorm orig="-9.83661600897224e-001" norm="0.000000"/><LinearNorm orig="2.79897885015328e+000" norm="1.000000"/></NormContinuous></DerivedField></NeuralInput><NeuralInput id="3"><DerivedField><NormContinuous field="ClimPC4"><LinearNorm orig="-2.01041447516216e+000" norm="0.000000"/><LinearNorm orig="1.63732366055998e+000" norm="1.000000"/></NormContinuous></DerivedField></NeuralInput><NeuralInput id="4"><DerivedField><NormContinuous field="ClimPC5"><LinearNorm orig="-2.25934363043270e+000" norm="0.000000"/><LinearNorm orig="2.01051219276550e+000" norm="1.000000"/></NormContinuous></DerivedField></NeuralInput><NeuralInput id="5"><DerivedField><NormContinuous field="PhenoPC1"><LinearNorm orig="-5.41067415675258e+000" norm="0.000000"/><LinearNorm orig="1.35699110945669e+000" norm="1.000000"/></NormContinuous></DerivedField></NeuralInput><NeuralInput id="6"><DerivedField><NormContinuous field="PhenoPC2"><LinearNorm orig="-1.66610794509485e+000" norm="0.000000"/><LinearNorm orig="1.69526550693665e+000" norm="1.000000"/></NormContinuous></DerivedField></NeuralInput></NeuralInputs><NeuralLayer numberOfNeurons="10" activationFunction="exponential"><Neuron id="7" bias="-6.76010754238150e-002"><Con from="0" weight="6.68320497735839e-001"/><Con from="1" weight="-1.77208567465908e-001"/><Con from="2" weight="-7.46770942882869e-001"/><Con from="3" weight="-2.40879294820333e+000"/><Con from="4" weight="7.10004409306447e-001"/><Con from="5" weight="1.74969786922824e+000"/><Con from="6" weight="3.96523596935970e-001"/></Neuron><Neuron id="8" bias="3.51304650763985e-001"><Con from="0" weight="-7.52105532998621e-001"/><Con from="1" weight="3.24083261108380e-001"/><Con from="2" weight="3.33054424464262e-001"/><Con from="3" weight="-4.46810776764888e-002"/><Con from="4" weight="-1.82842873845545e-001"/><Con from="5" weight="1.96107040370536e-001"/><Con from="6" weight="4.38346566092898e-001"/></Neuron><Neuron id="9" bias="3.90100176812069e-001"><Con from="0" weight="-7.33363566577205e-001"/><Con from="1" weight="2.63727252260829e-001"/><Con from="2" weight="2.92364772771950e-001"/><Con from="3" weight="2.68048879505033e-001"/><Con from="4" weight="-2.38720729993380e-001"/><Con from="5" weight="7.69874637823040e-002"/><Con from="6" weight="1.89477740338744e-002"/></Neuron><Neuron id="10" bias="5.40206833680070e-001"><Con from="0" weight="-1.14992939499567e+000"/><Con from="1" weight="3.72667101119439e-001"/><Con from="2" weight="6.03606167962294e-001"/><Con from="3" weight="7.50795236821320e-001"/><Con from="4" weight="-6.72548247477240e-001"/><Con from="5" weight="8.32073370400124e-002"/><Con from="6" weight="-8.24455526408236e-001"/></Neuron><Neuron id="11" bias="1.47995862324487e-001"><Con from="0" weight="-2.36013357617732e-001"/><Con from="1" weight="3.42112749141626e-002"/><Con from="2" weight="1.75126832523188e-001"/><Con from="3" weight="4.63464175662385e-001"/><Con from="4" weight="-6.16929217465198e-002"/><Con from="5" weight="-2.19479059443906e-001"/><Con from="6" weight="-2.27751614483623e-001"/></Neuron><Neuron id="12" bias="6.47724434664521e-002"><Con from="0" weight="-5.48249654075177e-002"/><Con from="1" weight="-5.60861861981239e-002"/><Con from="2" weight="-1.07582951181968e-001"/><Con from="3" weight="1.79409131085833e-001"/><Con from="4" weight="1.17612591562505e-001"/><Con from="5" weight="-1.99005780633799e-002"/><Con from="6" weight="3.47251895676683e-001"/></Neuron><Neuron id="13" bias="5.38554802556840e-002"><Con from="0" weight="8.71343555793938e-002"/><Con from="1" weight="-1.43796377796135e-001"/><Con from="2" weight="-1.58122065661083e-001"/><Con from="3" weight="-1.62934898510782e-002"/><Con from="4" weight="1.06550707468930e-001"/><Con from="5" weight="-8.88818274829853e-002"/><Con from="6" weight="2.83606153903499e-001"/></Neuron><Neuron id="14" bias="-1.63958419171359e-002"><Con from="0" weight="1.67107638613343e-001"/><Con from="1" weight="-2.26319937824352e-001"/><Con from="2" weight="-2.46485961604122e-001"/><Con from="3" weight="-1.20509713141538e-001"/><Con from="4" weight="2.61369920212944e-001"/><Con from="5" weight="4.57894001170861e-003"/><Con from="6" weight="5.03176785533112e-001"/></Neuron><Neuron id="15" bias="1.90003548380765e-001"><Con from="0" weight="-2.74753273733677e-001"/><Con from="1" weight="5.88178731289829e-002"/><Con from="2" weight="7.92831409936354e-002"/><Con from="3" weight="3.72893255783390e-001"/><Con from="4" weight="-1.08117028085555e-001"/><Con from="5" weight="-1.43780074009508e-001"/><Con from="6" weight="-1.65084124635455e-002"/></Neuron><Neuron id="16" bias="5.37545917772378e-002"><Con from="0" weight="-2.26418125485593e-001"/><Con from="1" weight="-3.78192185859478e-002"/><Con from="2" weight="1.38512624241292e-001"/><Con from="3" weight="5.28572242005961e-001"/><Con from="4" weight="-1.59602172067123e-001"/><Con from="5" weight="-2.44634025454269e-001"/><Con from="6" weight="-2.56969821454746e-001"/></Neuron></NeuralLayer><NeuralLayer numberOfNeurons="1" activationFunction="logistic"><Neuron id="17" bias="3.41691268424003e+000"><Con from="7" weight="2.60373435401101e-001"/><Con from="8" weight="-8.40412900240992e-001"/><Con from="9" weight="-6.09525333619231e-001"/><Con from="10" weight="-3.65944498379063e-001"/><Con from="11" weight="-4.78728739464009e-001"/><Con from="12" weight="-8.28417400870392e-001"/><Con from="13" weight="-5.74190517604463e-001"/><Con from="14" weight="-8.20251981902975e-001"/><Con from="15" weight="-5.37542805655830e-001"/><Con from="16" weight="-2.87499788793773e-001"/></Neuron></NeuralLayer><NeuralOutputs numberOfOutputs="1"><NeuralOutput outputNeuron="17"><DerivedField optype="continuous"><NormContinuous field="Sum_Humanother mammal_Start"><LinearNorm orig="0.00000000000000e+000" norm="0.00000000000000e+000"/><LinearNorm orig="4.00000000000000e+000" norm="1.00000000000000e+000"/></NormContinuous></DerivedField></NeuralOutput></NeuralOutputs></NeuralNetwork></PMML>
